# The role of the medial buttress plate in the treatment of Pauwels type II and III femoral neck fracture in nonelderly patients: a retrospective study and preliminary results

**DOI:** 10.1186/s12891-022-05056-8

**Published:** 2022-01-31

**Authors:** Chao Ma, Yanshi Liu, Jialin Liu, Li Chen, Jinyong Huang, Xuefeng Luo, Zengru Xie

**Affiliations:** 1 Department of Trauma Orthopedics, The First Affiliated Hospital of Xinjiang Medical University, Urumqi, China; 2 Department of Microrepair and Reconstruction, The First Affiliated Hospital of Xinjiang Medical University, Urumqi, China; 3 Department of Prosthodontics, The First Affiliated Hospital of Xinjiang Medical University, Urumqi, China; 4 Department of Ophthalmology, The First Affiliated Hospital of Xinjiang Medical University, Urumqi, China

**Keywords:** Multiple cannulated screws, Femoral neck fracture, Biomechanical microenvironment

## Abstract

**Background:**

The purpose of this study was to compare the effectiveness of multiple cannulated screws combined with medial buttress plate or not for the treatment of unstable femoral neck fracture in nonelderly patients.

**Methods:**

Sixty-nine nonelderly patients with Garden type III-IV femoral neck fracture were retrospectively analyzed. The patients were divided into MCS (multiple cannulated screws) group and CMBP (combined with medial buttress plate) group according to the surgical method. Patient’s demographic data, Harris Hip Score, EQ-5D index and complications at a minimum of 2 years follow-up were analyzed.

**Results:**

There were 47 patients in the MCS group (35 male and 12 females) with a mean age of 40.28 ± 12.64 years, whereas 22 patients in the CMBP group (17 male and 5 females) with a mean age of 43.86 ± 12.55 years. In the MCS group, there were 1 (2.1%) avascular necrosis, 5 (10.6%) postoperative nonunion, 5 (10.6%) implant failure, and 2 (4.3%) femoral neck shortening. While 1 (4.5%) implant failure, 2 (9.1%) postoperative nonunion and 2 (9.1%) impingement in the CMBP group. For patients with Pauwels type II and III femoral neck fracture, the CMBP group had higher HHS scores at 3 months after surgery than the MCS group (*P* < 0.05), whereas there was no statistical significance at 6 months, 1 year, and 2 years (*P* > 0.05). The same results were found in the EQ-5D index.

**Conclusions:**

In our cohort, we observed better outcomes in the CMBP group at 3 and 6 months, with later results similar between groups. However, there were fewer complications in the CMBP group, without obviously blood-supply disruption, especially in Pauwels type II and III. Further, anatomic reduction and stable fixation may contribute to satisfactory outcomes in the treatment of nonelderly displaced femoral neck fractures.

## Background

Femoral neck fractures in healthy nonelderly individuals are relatively uncommon and are usually caused by high-energy trauma [1]. Prompt diagnosis, anatomic reduction, and stable internal fixation may contribute the satisfactory outcomes [1–3]. However, the optimal definitive management of such fractures remains controversial and a challenging orthopaedic problem due to the complexity of anatomical relationship and a vulnerable blood supply to the femoral head, which could result in malunion, nonunion, or avascular necrosis [4–6].

The orthopaedic surgeon’s goals of obtaining and maintaining anatomic reduction until the bony union has been addressed by a number of surgical approaches and fixation constructs, including a sliding hip screw device, proximal femoral locking plates, cephalomedullary nails, and multiple cannulated parallel lag screws [7–9]. However, even with these treatment methods, complications are still common. Additionally, for all these injuries, there is no consensus on which single fixation option is superior [7]. Zhuang et al. concluded that avascular necrosis of femoral head and nonunion are major complications after the treatment of femoral neck fractures in young patients, and one of the important causes of complications is the insufficient stability provided by conventional implants [10]. Currently, it is widely recognized that displaced Pauwel type I and most type II fractures may be effectively managed with 3 parallel cancellous lag screws inserted in an inverted triangular configuration [11, 12]. Anthony et al. suggested that for Pauwel type III, basicervical, and highly comminuted unstable fracture patterns, sliding hip screw provides greater mechanical stability to resist the increased shear forces generated and should be used in place of cancellous screws [11]. Mir et al. proposed that anatomic reduction and fixation, coupled with a medial buttress plate that resists shearing forces, may reduce the historically high complication rate of vertical femoral neck fractures. This technique has not been widely applied to this injury pattern, and there is little published data demonstrating the outcomes [3].

The purpose of this study was to compare the effectiveness of multiple cannulated screws combined with medial buttress plate or not for the treatment of unstable femoral neck fracture in nonelderly patients.

## Methods

The retrospective study included 342 patients who underwent femoral neck fractures treated at our institution from April 2012 to February 2018.

The inclusion criteria were: (1) age between 18 to 65 years, (2) femoral neck fracture, Garden type III-IV, (3) fixation should be conducted less than 10 days from injury, (4) American Academy of Anesthesiology class 1 or 2, (5) no other fracture in the lower limb, (6) a minimum of 2 years follow-up. The exclusion criteria were (1) pathological fractures, (2) severe blood and immune system diseases, (3) severe multiple traumas or a previous history of ipsilateral hip or femur surgery, and (4) conditions such as osteoarthritis and post-dysplastic deformities. A total of 69 eligible patients were reviewed. There were 52 males and 17 females, with a mean age of 41.42 ± 12.63 years. Of all the patients, 47 patients were in MCS (multiple cannulated screws) group and 22 patients were in CMBP (combined with medial buttress plate) group. All patients gave written informed consent for their data to be published in our study. This study received approval from the Ethical Committee of our institution.

### Surgical procedures

Patients in both groups were under general anesthesia, lying supine on the operating table with the affected hip elevated at an angle of 10–15°. A C-arm x-ray machine was used to take anteroposterior and lateral radiographs of the femoral neck. The reduction was considered acceptable if the angle between the medial femoral head and the medial femoral shaft cortex in the anteroposterior and lateral radiographs (measured separately by two surgeons in our team) met the Garden Alignment Index Grade I criteria. Otherwise, the effectiveness of the reduction was considered unsatisfactory [13].

For patients in the MCS group, fixations were monitored under C-arm fluoroscopy and conducted with cannulated screws. The reduction was confirmed by biplane fluoroscopy, and a guide wire was drilled in the distal cortex of the greater trochanter abutting the calcar femorale, and the other two guide wires were drilled along the anteroposterior cortex, respectively, parallel to the first. The appropriate length cannulated screw is then drilled over the guide wire to a depth of 5 mm under the cartilage of the femoral head. Partially threaded 7.3 mm cannulated screws (WEGO) were used and implanted with an inverted triangle. Three cannulated screws were inserted parallel to each other [6, 14]. Open reduction was considered when anatomic reduction could not be achieved in the patient.

For patients in the CMBP group, the modified Smith-Petersen approach was used for this surgery. The anatomic reduction was achieved by a combination of manual traction and femoral head manipulation. In addition, the surgeon can reduce the femoral neck fracture under direct vision. After fluoroscopy confirmed the reduction, a small incision was made on the lateral side of the femur, through which the cannulated screw guide wires were drilled into the femoral head, and three cannulated screws were inserted parallel into the femoral head. The 1/3 tube buttress plate of AO was then shaped and placed on the anteromedial surface of the femoral neck and fixed with three screws [3, 10, 15].

### Postoperative management

Patients in both groups received perioperative antibiotics, as well as low molecular weight heparin (4000AxaIU) once daily for two weeks as antithrombotic prophylaxis. All patients began postoperative quadriceps muscle contraction and relaxation exercises on day 2, CPM (continuous passive motion) training on day 3. The patients were ambulated with axillary crutches and touch weight-bearing for six weeks, and partial weight-bearing for another six weeks. Regular follow-ups were performed at 1 month, 3 months, 6 months, 1 year, and 2 years after surgery, and instructions for mobilization, including weight-bearing as tolerated were provided based on the results of the radiological evaluation. At each follow-up, the degree of union, the position of the implant, and loss of fracture alignment were observed and femoral neck shortening was measured once on the anteroposterior and lateral radiographs by two physicians in our surgical team, respectively, ≥5 mm is considered a complication. At each time, the Harris Hip Score (HHS) [16] was calculated to evaluate the hip function, and the EQ-5D index (EuroQol five-dimension questionnaire) was used to measure the health-related quality of life. Nonunion was defined as a clearly visible fracture line one year postoperatively [15]. Avascular necrosis was identified by the Steinberg classification from stage 2 and upward [17].

All 69 patients’ demographic data, Harris Hip Score, EQ-5D index and complications at a minimum of 2 years follow-up were analyzed.

### Statistical analysis

Statistical analysis was performed using SPSS version 22.0 (SPSS Inc., Chicago, IL, USA). All quantitative data are presented as mean ± standard deviation (SD). Levene’s test was used to determine the distribution of data. The independent t-test for two samples was used to analyze the data. Categorical data were compared using the χ2 test or the Fisher exact probability method if the theoretical frequency was less than 1. A *P* value <0.05 was considered statistically significant.

## Results

The baseline data were shown in Table 1.

**Table 1:** Baseline characteristics of patients. Comparison between groups (mean ± standard deviation)

	MCS	CMBP	X^2^/t	*P*-value
Gender			0.063	0.801
Male	35	17		
Female	12	5		
Age (years)	40.28 ± 12.64	43.86 ± 12.55	−1.101	0.275
BMI	24.31 ± 2.90	24.53 ± 2.06	−0.319	0.751
Fractured limb			0.665	0.415
Left	22	8		
Right	25	14		
Mechanism of injuries			0.585	0.746
Traffic accident	33	14		
Fall	9	6		
Sport injury	5	2		
Garden classification			0.104	0.747
Type III	28	14		
Type IV	19	8		
Pauwels classification			19.127	*P* < 0.001
Type I	11	0		
Type II	29	8		
Type III	7	14		
ASA classification			0.153	0.696
Grade 1	32	16		
Grade 2	15	6		
Interval from injury to operation (d)	4.8 ± 1.6	4.9 ± 1.8	−0.176	0.861
Operative time (min)	52.17 ± 11.57	92.41 ± 17.87	−11.239	*P* < 0.001
Intraoperative blood loss (ml)	51.81 ± 19.21	265.23 ± 53.15	−24.408	*P* < 0.001

*MCS* multiple cannulated screws group, *CMBP* combined with medial buttress plate group, *BMI* body mass index, *ASA* American Society of Anesthesiologists

**Fig. 1:**
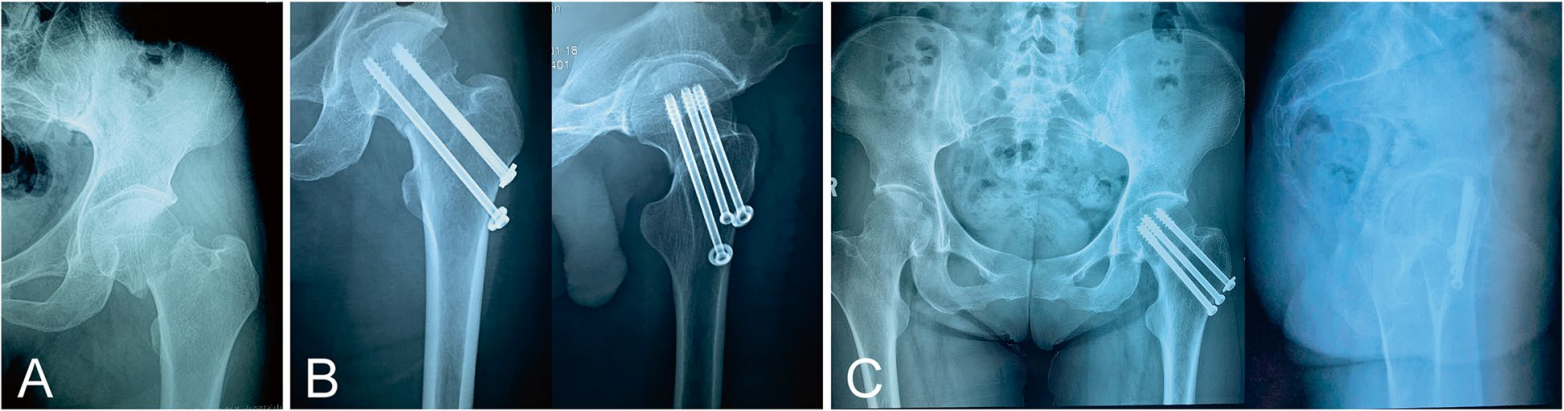
Example images of patient radiographs in the MCS group. **A** Preoperative. **B** Postoperative. **C** 1 year after surgery

**Fig. 2:**
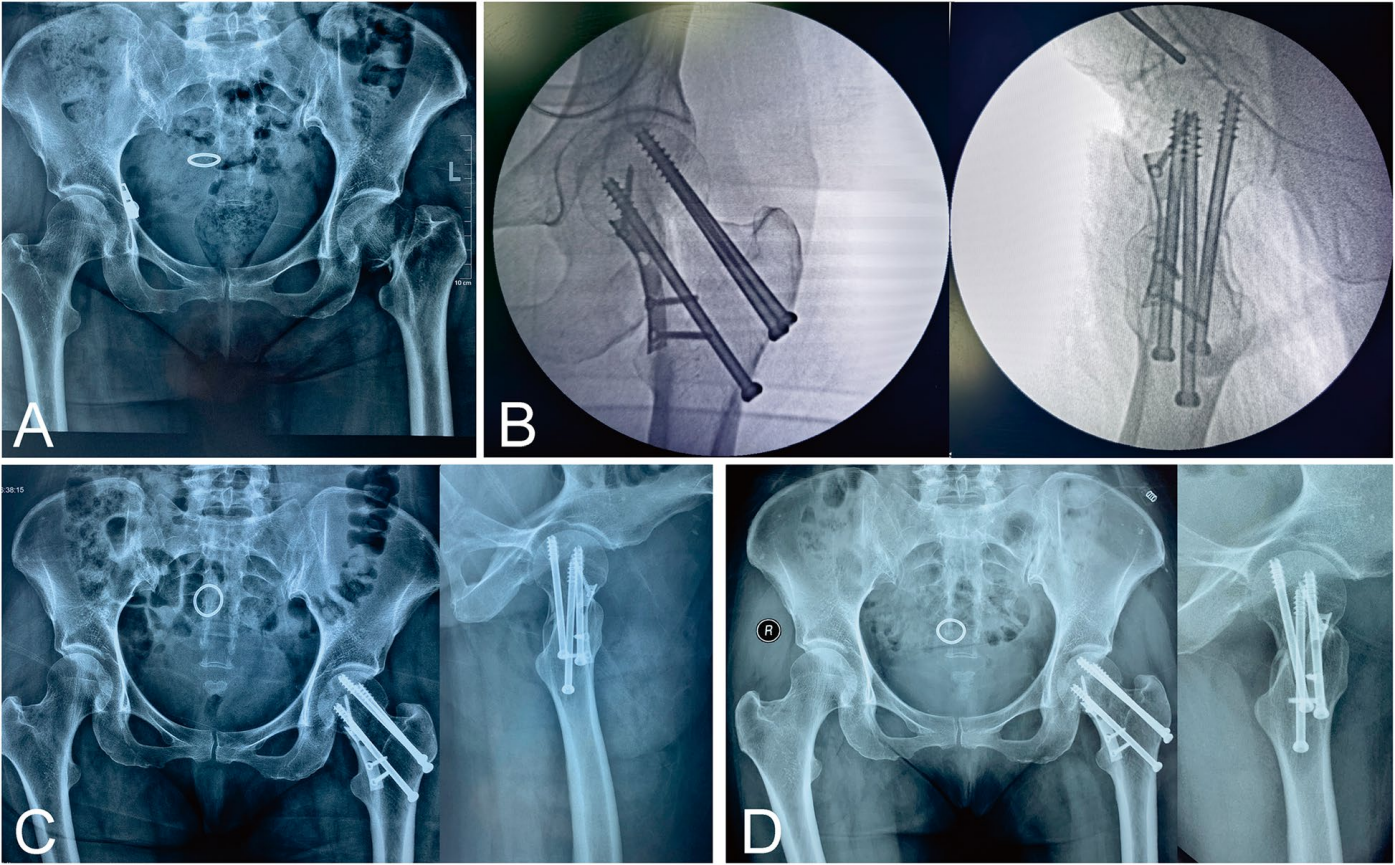
Example images of patient radiographs in the CMBP group. **A** Preoperative. **B** Intraoperative. **C** Postoperative. **D** 1 year after surgery

The mean elapsed time from injury to operation was 4.8 days (range 3 to 8 days) in MCS group, while 4.9 days (range 3 to 9 days) in CMBP group, the difference was not statistically significant (*P* > 0.05). 4 patients in MCS group underwent open reduction because of suboptimal reduction. Satisfactory reduction was achieved in all patients in both groups. The average operative time was 52.2 min (range 29 to 75 min) in MCS group and 92.4 min (range 74 to 134 min) in CMBP group. In addition, the mean intraoperative blood loss was 51.8 ml (range 20 to 100 ml) in MCS group and 265.2 ml (range 170 to 350 ml) in CMBP group, respectively. The differences in operative time and intraoperative blood loss between the two groups were statistically significant (*P* < 0.05). (Table 1). Example images of patient radiographs in both groups were shown in Figs. 1 and 2.

**Fig. 3:**
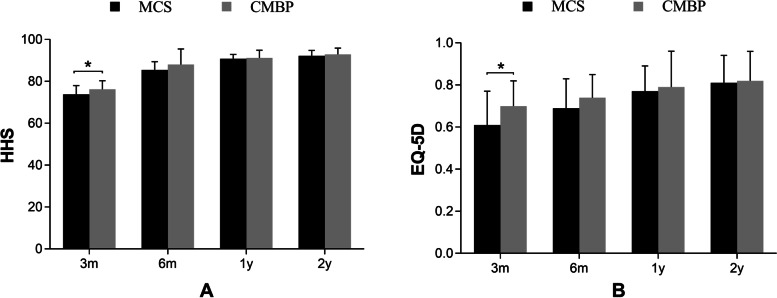
The HHS and EQ-5D of MCS and CMBP group (Pauwels type II and III, MCS: n = 36, CMBP: n = 22). MCS: multiple cannulated screws group. CMBP: combined with medial buttress plate group. * Significant difference between two groups (*p* < 0.05)

All patients in two groups were closely followed up at a minimum of two years after the operation (mean 2.3 years, range 2 to 3.6 years). As for the Harris Hip Scores and the score of EQ-5D index, the CMBP group had higher HHS scores at 3 months and 6 months after surgery than the MCS group (*P* < 0.05; 95% confidence interval (CI) = 0.34 to 4.71; 0.17 to 5.62), while no obvious difference at 1 year and 2 years (*P* > 0.05; 95% CI = 0.66 to 2.27; 1.07 to 1.86). (Table 2). At 6 months after surgery, excellent outcomes (HHS ≥ 90) were achieved in 6 (12.8%) and 10 (45.5%) cases in the MCS and CMBP group, respectively. The differences between MCS group and CMBP group in the treatment of unstable femoral neck fracture (Pauwels classification, Type II and Type III) were shown in Fig. 3. The CMBP group had higher HHS scores at 3 months after surgery than the MCS group (*P* < 0.05; 95% CI = 0.16 to 4.65), whereas there was no statistical significance at 6 months, 1 year, and 2 years (*P* > 0.05; 95% CI = 0.42 to 5.56; 1.05 to 1.95; 0.74 to 2.23). However, at 6 months after surgery, excellent outcomes (HHS ≥ 90) were achieved in 5 (13.9%) and 10 (45.5%) cases in the MCS and CMBP group, respectively. Similar results were found in the EQ-5D index.

**Table 2:** Functional outcome by HHS score and EQ-5D index. Comparison between groups (mean ± standard deviation)

Functional outcome	MCS (n = 47)	CMBP (n = 22)	t	*P*-value
HHS score				
3 months	73.66 ± 4.30	76.18 ± 4.13	−2.302	0.024
6 months	85.15 ± 3.92	88.05 ± 7.44	−2.123	0.037
12 months	90.43 ± 2.38	91.23 ± 3.66	−1.092	0.279
24 months	92.51 ± 2.80	92.91 ± 2.93	−0.543	0.589
EQ-5D index				
3 months	0.61 ± 0.16	0.70 ± 0.12	−2.385	0.020
6 months	0.68 ± 0.14	0.74 ± 0.11	−2.022	0.047
12 months	0.77 ± 0.11	0.79 ± 0.17	−0.792	0.431
24 months	0.81 ± 0.12	0.82 ± 0.14	−0.372	0.711

*MCS* multiple cannulated screws group, *CMBP* combined with medial buttress plate group, *HHS* Harris Hip Score, *EQ-5D* EuroQol five dimensions questionnaire

The incidence of complications related to the fracture is shown in Table 3. During the follow-up period in MCS group, 1 (2.1%) patient developed avascular necrosis. Postoperative nonunion occurred in 5 (10.6%) patients, 3 (6.4%) of these patients developed cannulated screw back out. Femoral neck shortening occurred in 2 (4.3%) cases in which cannulated screw back out and screw broken occurred in 1 (2.1%) case respectively, but the fracture appeared radiographically healed in a malunited position. 6 (12.8%) patients underwent hip arthroplasty in the second phase.

**Table 3:** Incidence of complications related to the fracture

Complications	MCS (n/N)	CMBP (n/N)
Avascular necrosis	1 (2.1%)	0 (0%)
Superficial or wound infection	0 (0%)	0 (0%)
Implant failure (cannulated screw back out/screw or plate breakage)	5 (10.6%)	1 (4.5%)
Nonunion	5 (10.6%)	2 (9.1%)
Impingement	0 (0%)	2 (9.1%)
Femoral neck shortening	2 (4.3%)	0 (0%)

*MCS* multiple cannulated screws group, *CMBP* combined with medial buttress plate group

In the CMBP group, nonunion was observed in 2 (9.1%) cases, of which 1 (4.5%) developed cannulated screw back out, and they were all managed by hip arthroplasty. 2 (9.1%) patients felt groin pain with deep hip joint flexion, because of impingement between the high position plate and acetabulum or labrum when the hip is in deep flexion, but 1(4.5%) resolved by 3 months post-operatively and the other 1 (4.5%) received removal of the medial buttress plate. There was no superficial or wound infection observed in both groups.

## Discussion

In order to compare the effectiveness of multiple cannulated screws combined with medial buttress plate or not in the treatment of unstable femoral neck fractures in non-elderly patients, this study retrospectively analyzed the clinical data of 69 elderly patients with Garden type III-IV femoral neck fractures.

Compressive stress, tensile stress and shear forces are generated at the proximal segment of the femur during force conduction, because of the femoral neck shaft angle. Stress trabecula bone, tension trabecula bone and calcar femorale are derived based on the loading and offered the ability of mechanotransduction. When a femoral neck fracture occurred, the normal stress conduction is lost because of these destroyed truss structures. Therefore, it is generally accepted that anatomic reduction with stable fixation offers patients the best biomechanical microenvironment needed for fracture healing without complication to maintain the native hip [18, 19].

The mechanical stability of fracture fixation structure is closely related to the implant, reduction quality as well as the structure and material characteristics of bone [9]. Kunapuli et al. [20] found that the medial femoral neck buttress plate enhanced the stability of cannulated screw and sliding hip screw constructs, significantly increased stiffness and load to failure. Giordano et al. [21] conducted a mechanical study to analyze the role of the medial buttress plate in Pauwels type III femoral neck fractures by comparing the resistance of two fixation methods using three cannulated screws, and the results showed that the medial buttress plate combined with multiple cannulated screw fixation provided a mechanically superior structure for Pauwels type III fractures. Additionally, Nwankwo et al. [19] conducted a mechanical test with ten matched pairs of young cadaveric femurs and concluded that the application of medial plates to Pauwels Type III femoral neck fractures significantly decreased angular displacement and shear.

In the present study, two patients (4.3%) suffered femoral neck shortening caused by implant failure in MCS group, while none was observed in CMBP group. Three cases (6.4%) developed postoperative nonunion with cannulated screw back out in MCS group and one (4.5%) in CMBP group. Nonunion is the main complication following internal fixation of the femoral neck fractures [22]. The 91% union rate in CMBP group is favorable compared to the study for Pauwels type III fractures, in which union rates ranging from 73.7 to 89% [5, 15, 23, 24].

We speculate that the satisfactory outcomes probably derived from anatomic reduction under the direct vision and the stable fixation under the combination of intramedullary and extramedullary in the CMBP group. Thus, there is a potential benefit that combines a medial buttress plate with cannulated screws in the anteromedial region of the femur neck improves the mechanical resistance of fixation in unstable fractures, especially in Pauwels type II and type III.

Preservation of the blood supply to the femoral head is most important in the treatment of femoral neck fractures. The medial circumflex artery (MFCA) is the main vascular supply to the femoral head, and the superior retinacular artery (SRA) is the most important branch [25–27]. The SRA and its branches are closely located to the femoral neck and are easily destroyed in displaced femoral neck fractures [26, 28]. It has also been shown that the inferior retinacular artery (IRA) also plays an important role in femoral head perfusion [25, 29]. IRA was present in all hips and the point of entry into the hip capsule as well as the intra-articular course were consistent. The study by Sara et al. [27] determined the anatomical (especially in vascular) feasibility of this approach to the treatment of femoral neck fractures via an anterior approach to the hip. They described the location of the vessels around the femoral neck by using a clock-face system, with the IRA coursing posteromedially between 7:00 and 8:00, placing a straight plate medially or slightly anteriorly will not disrupt the IRA. In addition, the lesser trochanter can be used as a positioning reference and the safe zone for placing the straight plate is just anterior to the lesser trochanter (5:00–6:00) when IRA is less well-visualized. The results of these studies not only provided an anatomical basis for the application of the medial femoral neck plate, but will also change the misconception that the plate would disrupt the blood supply to the femoral head.

In the present study, for patients in the CMBP group, the 1/3 tube buttress plate of AO was placed anteromedially at the femoral neck via a modified Smith-Petersen approach, which is consistent with the recommendations of Sara et al. [27]. Compared with the MCS group, open reduction allowed emptying of the intra-articular hematoma, reducing the risk of avascular necrosis of the femoral head due to high intra-articular pressure [30]. In addition, the femur is usually slightly externally rotated during the procedure to aid in visualization of the femoral neck. At this time, the Weibrecht’s ligament and the IRA that travels within are naturally fall away from the femoral neck, reducing the risk of damaging the IRA during the placement of the plate. During the follow-up, the absence of avascular necrosis in the CMBP group supports that this surgical approach does not increase the osteonecrosis rate of Garden type III-IV fractures. On the other hand, postoperative nonunion is probably related to the blood supply, 2 (4.3%) patients in the MCS group and 1 (4.5%) in the CMBP group experiencing nonunion excluding implant failure. This supports that the medial buttress plate does not increase the nonunion rate.

As shown in Table 1, there is no doubt that the CMBP group has a longer operation time and more intraoperative blood loss. This is acceptable given the benefits that the medial buttress plate brings to the patient in terms of maintenance of fracture stability. As shown in Table 2, thanks to the stability of the fracture, HHS, and EQ scores were higher and functional recovery of patients was earlier in the CMBP group at 3 and 6 months. What should be noticed is that, similar to the results reported by Lucas [18], 2 patients in the CBMP group had impingement with hip flexion, which is probably due to the plate being too proximal.

There are some limitations to our study. First, given that this is retrospective data from the same institution with relatively small sample size, a prudent attitude should be adopted regarding the interpretations of our outcomes. Second, to get more persuasive and generalizable results, more comprehensive studies should be conducted, such as multicenter trials with large sample sizes.

## Conclusions

In our cohort, we observed better outcomes in the CMBP group at 3 and 6 months, with later results similar between groups. However, there were fewer complications in the CMBP group, without obviously blood-supply disruption, especially in Pauwels type II and III. Further, anatomic reduction and stable fixation may contribute to satisfactory outcomes in the treatment of nonelderly displaced femoral neck fractures.

## Data Availability

The datasets analyzed during the current study are available from the corresponding author on reasonable request.

## Abbreviations

MCS: Multiple cannulated screws
CMBP: Combined with medial buttress plate
CPM: Continuous passive motion
HHS: The Harris Hip Score
EQ-5D: EuroQol five-dimension questionnaire
SD: Standard deviation
MFCA: The medial femoral circumflex artery
SRA: Superior retinacular artery
IRA: Inferior retinacular artery
